# Supplementary material for: Reduced Arbuscular Mycorrhizal Fungi (AMF) Diversity in Light and Moderate Fire Sites in Taiga Forests, Northeast China
Source: Microorganisms. 2023 Jul 19;11(7):1836. doi: 10.3390/microorganisms11071836 (PMC10385377; doi:10.3390/microorganisms11071836)
Supplement: Supplementary file 1 [file microorganisms-11-01836-s001.zip › microorganisms-2496341-supplementary.pdf]

Supplementary Materials

Table S1. Analysis of the variability of genus level abundance in fire sites of different intensities.

Genus Name	CK(Control-Blank)	L(Light-Fire)	M(Moderate-Fire)	H(Haevy-Fire)
Glomus	0.95 ± 0.06a	0.08 ± 0.15b	0	0.92 ± 0.03a
unclassified_c__Glomeromycetes	0	0.81 ± 0.19a	0.71 ± 0.07a	0.07 ± 0.03b
Paraglomus	0	0.07 ± 0.11a	0.01 ± 0.02a	0
unclassified_f__Archaeosporaceae	0	0.04 ± 0.06a	0.14 ± 0.12a	0.01 ± 0.00a
Ambispora	0.01 ± 0.02a	0	0.14 ± 0.12a	0
Acaulospora	0.04 ± 0.07a	0	0	0

Different letters within a row indicate significant differences ($p < 0.05$).
